# The Relationship Between Job Satisfaction, Life Satisfaction, and Optimism Among Nursing Staff: A Cross-Sectional Study

**DOI:** 10.1155/jonm/7054010

**Published:** 2025-11-11

**Authors:** Stanisław Manulik, Maria Jędrzejczyk, Michał Czapla, Ercole Vellone, Izabella Uchmanowicz

**Affiliations:** ^1^Department of Nursing, Faculty of Nursing and Midwifery, Wroclaw Medical University, Wroclaw, Poland; ^2^Division of Scientific Research and Innovation in Emergency Medical Service, Department of Emergency Medical Service, Faculty of Nursing and Midwifery, Wroclaw Medical University, Wroclaw, Poland; ^3^Group of Research in Care (GRUPAC), Faculty of Health Science, University of La Rioja, Logroño, Spain; ^4^Nursing Care and Education Research Group (GRIECE), Faculty of Nursing and Podology, University of Valencia, Valencia, Spain; ^5^Department of Biomedicine and Prevention, University of Rome Tor Vergata, Rome, Italy; ^6^Centre for Cardiovascular Health, Edinburgh Napier University, Sighthill Campus, Edinburgh, UK

**Keywords:** job satisfaction, life satisfaction, midwifery, nursing, optimism

## Abstract

**Background:**

Job and life satisfaction, alongside optimism, are critical determinants of psychological resilience in frontline healthcare staff. This study aims to examine the key predictors of job satisfaction, life satisfaction, and optimism among nursing and midwifery professionals in Poland.

**Methods:**

A cross-sectional study was conducted among 475 nurses and midwives employed in various healthcare settings in Poland. Data were collected through an anonymous online questionnaire including demographic and occupational items and three validated scales: Satisfaction With Job Scale (SJS), Satisfaction With Life Scale (SWLS), and Life Orientation Test–Revised (LOT-R).

**Results:**

The findings revealed significant variations in job satisfaction, life satisfaction, and optimism among nurses and midwives. The mean job satisfaction score (SJS) was 20.71 (SD = 6.9), indicating a moderate level of satisfaction. Life satisfaction (SWLS) had a mean score of 21.33 (SD = 6.61), while optimism (LOT-R) was 14.85 (SD = 3.97), suggesting variability in positive expectations for the future among the participants. Job satisfaction was negatively associated with shift-based work (*β* = −1.71, *p*=0.013), salary dissatisfaction (*β* = −3.828, *p* < 0.001), and higher stress levels (*β* = −0.127, *p* < 0.001). Life satisfaction was positively associated with being in a relationship (*β* = 3.58, *p* < 0.001) and a higher patient load (*β* = 1.969, *p*=0.049). Optimism was positively predicted by higher education (*β* = 3.797, *p*=0.015) and working in long-term care (*β* = 2.167, *p*=0.004) but decreased with increasing stress (*β* = −0.063, *p*=0.001).

**Conclusion:**

Job satisfaction, life satisfaction, and optimism among nursing staff are significantly influenced by salary, stress levels, work environment, and education. Interventions aimed at improving working conditions, reducing stress, and promoting psychological resilience may enhance staff well-being and, consequently, patient care quality.

## 1. Introduction

Job satisfaction and life satisfaction are key elements of healthcare professionals' well-being, particularly among nurses and midwives [1]. These professions are associated with emotional and physical strain, shift work, and high patient loads, all of which may lead to occupational stress, burnout, and reduced professional engagement [2, 3]. Understanding the interplay between job satisfaction, overall life satisfaction, and optimism is essential for enhancing nurses' resilience and improving care quality [4]. The existing research highlights that job satisfaction is closely linked to factors such as workload, salary, work environment, and autonomy in decision-making [5]. Dissatisfaction in these areas can lead to lower motivation, increased stress, and reduced quality of patient care [6]. Similarly, life satisfaction, which reflects an individual's overall well-being and perception of life fulfillment, is influenced by both professional and personal factors, including job conditions, social relationships, and financial stability [7, 8]. Furthermore, optimism, as a psychological resource, plays a pivotal role in coping with occupational stress and maintaining a positive outlook on career and life [9].

Despite growing interest in these relationships, limited studies have explored how job satisfaction, life satisfaction, and optimism interact specifically within the nursing and midwifery professions in Poland. Additionally, the impact of external stressors, such as economic instability and geopolitical events, has yet to be fully examined in this context. Given the significant proportion of nurses working under challenging conditions—including shift work, high patient loads, and multiple job commitments—there is a need for a deeper understanding of the determinants of their job satisfaction and overall well-being.

This exploratory study aims to examine the key predictors of job satisfaction, life satisfaction, and optimism among nursing and midwifery professionals in Poland. By analyzing the impact of demographic and occupational variables, as well as external stressors, we seek to provide evidence-based recommendations for improving working conditions and supporting the mental well-being of healthcare personnel.

## 2. Materials and Methods

### 2.1. Study Design and Participants

This was a cross-sectional exploratory study conducted among 475 nurses and midwives employed in various healthcare and related settings in Wrocław, Poland, and surrounding areas. In Poland, nursing and midwifery are regulated as two separate professions. In some countries (e.g., Spain and several other European states), midwifery functions as a postregistration nursing specialization; nonetheless, for the purposes of this study, both groups were analyzed jointly because they share similar clinical settings, psychosocial stressors, and ethical responsibilities. Given the relatively small proportion of midwives in our sample (approximately 11%), conducting separate analyses would likely have resulted in limited statistical power and unstable estimates. Participants were recruited through convenience sampling, with inclusion criteria including at least 1 year of professional experience, consent to participate, and completion of an online survey. Strengthening the Reporting of Observational Studies in Epidemiology (STROBE) guidelines were followed.

### 2.2. Data Collection

The study utilized a structured questionnaire composed of demographic and occupational characteristics and three standardized psychometric scales to assess job satisfaction, life satisfaction, and optimism. Data were collected via an anonymous online questionnaire distributed among nurses and midwives working in hospitals and outpatient clinics in Wrocław and the surrounding Lower Silesian region. Each participant received an individualized survey link generated on a secure institutional server, which prevented multiple submissions from the same device. No personally identifiable or sensitive data were collected. Before starting the survey, participants read an electronic information sheet and provided informed consent. They could withdraw from the study at any time before submitting the questionnaire. Participation was voluntary, and the data collection took place between June and September 2024. Only fully completed questionnaires from respondents who confirmed current employment as nurses or midwives were included in the final analysis.

### 2.3. Research Tools

Three validated psychometric instruments were used in this study: the Satisfaction with Job Scale (SJS), the Satisfaction With Life Scale (SWLS), and the Life Orientation Test–Revised (LOT-R). All tools were used in their Polish versions, validated for the target population.

The SJS, developed by Zalewska, is a unidimensional tool designed to assess the cognitive aspect of overall job satisfaction. The scale includes five statements related to general satisfaction with one's work. Each item is rated on a seven-point Likert scale, where 1 corresponds to “*strongly disagree*” and 7 to “*strongly agree*.” The total score ranges from 5 to 35, with higher scores indicating greater job satisfaction. The Polish version has demonstrated good internal consistency, with Cronbach's alpha ranging from 0.752 to 0.864, depending on the study population [10].

The SWLS was developed by Diener et al. to measure global cognitive assessment of life satisfaction [11]. It was adapted into Polish by Juczyński. It consists of five items that reflect a person's global evaluation of their life satisfaction. Each item is scored on a seven-point Likert scale, ranging from 1 (“*strongly disagree*”) to 7 (“*strongly agree*”). The total score ranges from 5 to 35, with higher scores reflecting greater life satisfaction. The Polish version of the SWLS has shown good reliability (Cronbach's alpha = 0.81) and theoretical validity, demonstrated through positive correlations with self-esteem and emotional control, and negative correlations with perceived stress [12].

The LOT-R was originally developed by Carver et al. to assess dispositional optimism [13]. The Polish adaptation was developed by Juczyński. The LOT-R contains 10 items, including six diagnostic statements (three positively and three negatively worded) and four fillers. Each item is rated on a five-point Likert scale from 0 (“*strongly disagree*”) to 4 (“*strongly agree*”). The final score, ranging from 0 to 24, is calculated based on the six diagnostic items, with higher scores indicating greater optimism. The Polish version has shown good psychometric properties, with Cronbach's alpha of 0.76. Its construct validity is supported by correlations with self-esteem and positive emotionality [12].

### 2.4. Statistical Analysis

Descriptive statistics (mean, standard deviation, median, and quartiles) were used to summarize quantitative variables, while categorical variables were presented as frequencies and percentages. To identify predictors of job satisfaction, life satisfaction, and optimism, linear regression models (both univariate and multivariate) were applied. Variables that showed significant or near-significant effects in univariate analyses (*p* < 0.2) were included in the multivariate regression models to adjust for potential confounders. Statistical significance was set at *p* < 0.05. All statistical analyses were conducted using the R statistical software (Version 4.4.2).

## 3. Results

The sociodemographic characteristics of the 475 participating nurses and midwives are summarized in Table 1. The sample was predominantly female and middle-aged, with most participants holding higher education degrees and working in inpatient care settings, often in shift-based systems. Nearly half reported monthly earnings above 1400 EUR, yet salary dissatisfaction remained common among respondents.

Table 2 summarizes the standardized psychometric measurements assessing job satisfaction (SJS), life satisfaction (SWLS), and optimism (LOT-R) among the 475 participants. On average, participants reported moderate levels of job and life satisfaction and a neutral to moderately optimistic outlook (Table 2). Additionally, participants rated their perceived stress level using a single-item question on a five-point Likert scale, the results of which were included in subsequent analyses.

Table 3 presents the results of univariate and multivariate linear regression analyses identifying predictors of job satisfaction (SJS scores) among nurses and midwives. In the univariate analysis, several occupational and psychosocial factors—including shift-based work schedules, hospital employment, salary dissatisfaction, and higher perceived stress levels (assessed with a single-item question)—were negatively associated with job satisfaction. After adjustment for multiple variables, working in outpatient care remained a positive predictor, whereas salary dissatisfaction and perceived stress showed the strongest negative associations.

Table 4 summarizes the results of regression analyses identifying factors influencing dispositional optimism (LOT-R scores). Higher education, specialization training, and employment in long-term care were associated with greater optimism, while salary dissatisfaction and elevated stress levels were significant negative predictors. These findings highlight the strong interplay between job-related factors, psychological well-being, and professional environment in nursing and midwifery practice.

## 4. Discussion

The findings of this study provide valuable insights into the relationships between job satisfaction, life satisfaction, optimism, and workplace factors among nurses and midwives. Several key determinants of well-being were identified, emphasizing the need for organizational improvements in healthcare settings.

The study confirmed that job satisfaction (SJS score) is significantly influenced by salary dissatisfaction, work system, and stress levels. Nurses and midwives working in shift-based schedules reported lower satisfaction, aligning with previous research indicating that irregular work hours contribute to fatigue, work-life imbalance, and increased stress. Furthermore, working in inpatient healthcare settings was associated with lower job satisfaction, possibly due to higher patient loads, limited autonomy, and emotional burden from long-term patient care [14, 15].

On the other hand, nurses working in outpatient care reported higher satisfaction, potentially due to greater autonomy, lower work intensity, and reduced emotional strain. These findings emphasize the need for healthcare institutions to address workload distribution, scheduling flexibility, and support systems for shift workers [16, 17].

The strongest negative predictor of job satisfaction was salary dissatisfaction, which is consistent with previous studies showing that inadequate compensation leads to burnout, turnover, and job dissatisfaction [18, 19]. Notably, those uncertain about their salary satisfaction also exhibited lower optimism, suggesting that financial instability is a key stressor in this professional group [20].

Given that a significant proportion of participants worked multiple jobs, it is likely that nurses take on additional employment to compensate for low wages, contributing to physical and emotional exhaustion [21, 22]. Addressing salary concerns through fair remuneration, financial incentives, and career progression opportunities is crucial for improving job satisfaction and staff retention.

Optimism (LOT-R score) was significantly influenced by workplace setting, education level, salary satisfaction, and stress levels. Higher education and professional specialization were associated with increased optimism, indicating that ongoing professional development enhances nurses' self-efficacy, career satisfaction, and overall positive orientation [7, 23].

Working in long-term care settings was linked to higher optimism, possibly due to structured work environments and sustained patient relationships, fostering a sense of accomplishment and professional fulfillment. Conversely, nurses working in nonsurgical (conservative) wards reported lower optimism, potentially due to higher workloads, emotional burdens, and limited procedural diversity, consistent with the findings from previous studies highlighting how resource-limited settings contribute to reduced optimism and increased nursing care rationing [7]. Caring for 6–15 patients per shift correlated positively with optimism, suggesting that optimal patient load facilitates effective time management and job satisfaction, aligning with the findings from multicenter studies indicating the importance of workload balance in reducing care rationing and enhancing psychological well-being among nurses [24].

Stress emerged as a significant negative predictor of both job satisfaction and optimism, with each incremental stress point adversely affecting these outcomes. These findings resonate with Maslach and Leiter's burnout model, emphasizing how emotional exhaustion, depersonalization, and reduced personal accomplishment detrimentally impact job satisfaction [25]. Additionally, Uchmanowicz et al. found strong correlations between burnout, lower life satisfaction, and increased nursing care rationing, emphasizing the necessity of proactive stress reduction strategies to improve care quality and nurse well-being [26].

Broader geopolitical stressors, such as the ongoing war in Ukraine, may additionally contribute to psychological strain among healthcare professionals in the region. Although this study did not directly assess such factors, they highlight the urgency of implementing mental health support programs and resilience training within healthcare organizations. These initiatives can enhance nurses' optimism and buffer against external psychological stressors, promoting healthier work environments and reducing implicit nursing care rationing [7].

Finally, leadership modeling and positive orientation training are recommended as practical nursing management interventions to cultivate optimism, enhance job satisfaction, and minimize nursing care rationing [7, 24, 27, 28].

### 4.1. Limitations

This study has several limitations that should be acknowledged. First, the cross-sectional design limits the ability to establish causal relationships between job satisfaction, life satisfaction, optimism, and associated predictors. Longitudinal studies are recommended to assess the dynamics of these variables over time. Second, the use of self-reported questionnaires may introduce response biases, including social desirability and recall bias. Third, although the sample was diverse in terms of age, workplace setting, and experience, it was limited to nursing staff in Poland, with most participants recruited from Wrocław and surrounding areas. Therefore, generalizability to other regions or countries may be limited. Additionally, nurses and midwives were analyzed together due to the relatively small number of midwives in the sample; while justified by their shared professional context, future research should aim for a more balanced representation to enable between-profession comparisons. Finally, data collection was conducted through secure online links distributed via professional training centers; the use of an online survey may still carry a potential risk of self-selection bias and limited control over participants' response environments. Future studies should consider more comprehensive tools for assessing geopolitical stressors and their long-term effects on healthcare personnel.

## 5. Conclusions

This study demonstrates that nurses' optimism and job satisfaction are significantly influenced by financial stability, workplace conditions, stress levels, and educational opportunities. Salary dissatisfaction was the strongest negative predictor of job satisfaction, emphasizing ongoing financial concerns among nurses. Structured workplace environments, particularly those with optimal patient loads enhanced optimism by facilitating effective time management and reducing nursing care rationing. Conversely, high-stress settings negatively impacted optimism due to workload, emotional strain, and limited procedural variety.

Higher education and specialization positively correlated with increased optimism, suggesting that professional development is essential for career satisfaction and self-efficacy. Stress, exacerbated by financial insecurity and geopolitical instability, remains a significant barrier to nurses' psychological well-being. Targeted interventions, including competitive salaries, structured peer support, professional development opportunities, and resilience training, are essential for fostering optimism, enhancing job satisfaction, and improving overall healthcare quality.

### 5.1. Relevance to Clinical Practice

Workplace satisfaction and optimism are essential elements of healthcare professionals' resilience and retention. This study highlights that structured work environments, financial stability, and reduced stress are associated with greater job satisfaction and optimism among nurses and midwives. These insights can inform management and policy strategies to enhance healthcare staff well-being, reduce turnover, and ultimately improve patient care outcomes.

## Figures and Tables

**Table 1:** Demographic and occupational characteristics of the study population.

Parameter		*N* = 475
Sex	Female	460 (96.84%)
	Male	15 (3.16%)

Age	< 30 years	73 (15.37%)
	31–40 years	69 (14.53%)
	41–50 years	148 (31.16%)
	51–60 years	167 (35.16%)
	> 60 years	18 (3.79%)

Education	Medical high school	54 (11.37%)
	Medical vocational school	38 (8.00%)
	Bachelor's in nursing/midwifery	164 (34.53%)
	Master's in nursing/midwifery	212 (44.63%)
	Other	7 (1.47%)

Postgraduate education^∗^	Qualification courses	296 (62.32%)
	Specialist courses	313 (65.89%)
	Specialization	309 (65.05%)

Place of residence	Village	147 (30.95%)
	City ≤ 50,000	116 (24.42%)
	City 50,001–100,000	55 (11.58%)
	City 100,001–250,000	40 (8.42%)
	City > 250,000	117 (24.63%)

Marital status	Single	80 (16.84%)
	In a relationship	383 (80.63%)
	Widowed	12 (2.53%)

Profession	Nurse	423 (89.05%)
	Midwife	52 (10.95%)

Number of jobs	1	232 (48.84%)
	2	152 (32.00%)
	3	50 (10.53%)
	4	9 (1.89%)
	5	5 (1.05%)
	More than 5	27 (5.68%)

Work setting^∗^	Outpatient care	168 (35.37%)
	Inpatient care	335 (70.53%)
	Nursing home	10 (2.11%)
	Long-term care	30 (6.32%)
	Trade union organization	6 (1.26%)
	University or college	25 (5.26%)
	Other	34 (7.16%)

Work schedule	Single-shift	194 (40.84%)
	Shift-based	281 (59.16%)

Average number of patients per shift	1–5	83 (17.47%)
	6–10	106 (22.32%)
	11–15	76 (16.00%)
	16–25	95 (20.00%)
	26–35	45 (9.47%)
	> 36	70 (14.74%)

Years of experience	1–5 years	68 (14.32%)
	6–10 years	51 (10.74%)
	11–15 years	53 (11.16%)
	16–20 years	33 (6.95%)
	> 21 years	270 (56.84%)

Ward profile^∗^	Conservative	164 (34.53%)
	Surgical	168 (35.37%)
	Operating theater	8 (1.68%)
	Intensive care unit	60 (12.63%)
	Pediatric	31 (6.53%)
	Primary care	11 (2.32%)
	Emergency department	20 (4.21%)
	Other	74 (15.58%)

Working hours per month	< 100 h	24 (5.05%)
	101–200 h	331 (69.68%)
	201–300 h	107 (22.53%)
	> 301 h	13 (2.74%)

Employment form^∗^	Employment contract	439 (92.42%)
	Individual nursing practice	33 (6.95%)
	Contract	30 (6.32%)
	Civil contracts (mandate/commission)	136 (28.63%)
	Other	4 (0.84%)

Net monthly income	< 230 EUR	4 (0.84%)
	230–460 EUR	6 (1.26%)
	461–700 EUR	6 (1.26%)
	701–930 EUR	30 (6.32%)
	931–1160 EUR	70 (14.74%)
	1161–1400 EUR	131 (27.58%)
	> 1400 EUR	228 (48.00%)

Salary satisfaction	Yes	193 (40.63%)
	No	235 (49.47%)
	Undecided	47 (9.89%)

^∗^Multiple choice: percentages do not add up to 100.

**Table 2:** Standardized measurement tools (SJS, SWLS, and LOT-R).

Parameter		Total (*N* = 475)
SJS	Mean (SD)	20.71 (6.9)
	Median (IQR)	22 (16–25)
	Range	5–35
	*N*	475

SWLS	Low sense of satisfaction with life	123 (25.89%)
	Average sense of satisfaction with life	160 (33.68%)
	High sense of satisfaction with life	192 (40.42%)
	Mean (SD)	21.33 (6.61)
	Median (IQR)	22 (17–26)
	Range	5–35
	*N*	475

LOT-R	Pessimistic	138 (29.05%)
	Neutral	181 (38.11%)
	Optimistic	156 (32.84%)
	Mean (SD)	14.85 (3.97)
	Median (IQR)	15 (12–18)
	Range	1–24
	N	475

*Note:* SJS = Satisfaction with Job Scale and IQR = interquartile range.

Abbreviations: LOT-R = Life Orientation Test-Revised, SD = standard deviation, and SWLS = Satisfaction With Life Scale.

**Table 3:** Predictors of job satisfaction (SJS score).

Variable		Univariate models				Multivariate model			
		Parameter	95% CI		*p*	Parameter	95% CI		*p*
Sex	Female	Ref.							
	Male	1.606	−1.952	5.163	0.376				

Age	< 30 years	Ref.				Ref.			
	31–40 years	0.262	−2.001	2.525	0.82	0.393	−1.767	2.553	0.721
	41–50 years	−0.325	−2.252	1.603	0.741	−0.336	−2.258	1.586	0.732
	51–60 years	−2.107	−3.998	−0.216	0.029^∗^	−1.277	−3.211	0.657	0.195
	> 60 years	−0.132	−3.678	3.415	0.942	−0.242	−3.746	3.263	0.892

Education	Medical high school	Ref.				Ref.			
	Medical vocational school	1.897	−0.95	4.743	0.191	1.697	−1.082	4.476	0.231
	Bachelor's in nursing/midwifery	1.389	−0.72	3.498	0.196	0.67	−1.364	2.703	0.518
	Master's in nursing/midwifery	3.135	1.085	5.184	0.003^∗^	1.81	−0.252	3.873	0.085
	Other	3.513	−1.887	8.913	0.202	2.933	−2.212	8.079	0.263

Qualification courses	No	Ref.							
	Yes	−0.535	−1.819	0.749	0.414				

Specialist courses	No	Ref.							
	Yes	0.649	−0.663	1.961	0.332				

Specialization	No	Ref.							
	Yes	0.196	−1.11	1.501	0.769				

Place of residence	Village	Ref.				Ref.			
	City ≤ 50,000	−0.085	−1.77	1.599	0.921	−0.136	−1.731	1.459	0.867
	City 50,001–100,000	0.055	−2.089	2.199	0.96	−0.256	−2.261	1.749	0.802
	City 100,001–250,000	0.873	−1.545	3.292	0.478	0.773	−1.552	3.098	0.514
	City > 250,000	1.323	−0.357	3.004	0.123	0.618	−0.986	2.223	0.449

Marital status	Single	Ref.							
	In a relationship	−0.463	−2.131	1.204	0.585				
	Widowed	−2.567	−6.766	1.632	0.23				

Profession	Nurse	Ref.				Ref.			
	Midwife	−1.814	−3.801	0.173	0.074	−2.063	−4.008	−0.118	0.038^∗^

Number of jobs	1	Ref.							
	2	0.119	−1.302	1.54	0.869				
	3	−0.336	−2.459	1.787	0.756				
	4	0.113	−4.513	4.739	0.962				
	5	−0.376	−6.53	5.779	0.905				
	More than 5	−1.146	−3.915	1.622	0.416				

Outpatient care	No	Ref.				Ref.			
	Yes	2.491	1.208	3.774	< 0.001^∗^	2.166	0.603	3.729	0.007^∗^

Inpatient care	No	Ref.				Ref.			
	Yes	−1.959	−3.313	−0.604	0.005^∗^	−0.119	−1.836	1.598	0.892

Nursing home	No	Ref.							
	Yes	0.09	−4.247	4.428	0.967				

Long-term care	No	Ref.							
	Yes	−0.439	−2.999	2.12	0.736				

Trade union organization	No	Ref.				Ref.			
	Yes	4.681	−0.879	10.24	0.099	4.299	−0.891	9.49	0.104

University or college	No	Ref.							
	Yes	0.347	−2.442	3.135	0.807				

Other	No	Ref.							
	Yes	−1.463	−3.875	0.948	0.234				

Work schedule	Single-shift	Ref.				Ref.			
	Shift-based	−1.577	−2.835	−0.318	0.014^∗^	−1.71	−3.061	−0.358	0.013^∗^

Average number of patients per shift	1–5	Ref.				Ref.			
	6–10	−0.212	−2.201	1.777	0.834	0.026	−1.93	1.983	0.979
	11–15	−1.463	−3.618	0.692	0.183	−0.223	−2.458	2.012	0.844
	16–25	−1.184	−3.224	0.855	0.254	−0.794	−2.94	1.352	0.467
	26–35	−1.809	−4.321	0.704	0.158	−1.777	−4.299	0.744	0.167
	> 36	−0.956	−3.159	1.246	0.394	−0.483	−2.838	1.871	0.687

Years of experience	0–5 years	Ref.							
	6–10 years	−0.5	−3.007	2.007	0.695				
	11–15 years	1.052	−1.429	3.532	0.405				
	16–20 years	−1.231	−4.102	1.641	0.4				
	> 21 years	−1.142	−2.978	0.695	0.223				

Conservative	No	Ref.				Ref.			
	Yes	−0.947	−2.254	0.36	0.155	−0.466	−1.783	0.85	0.487

Surgical	No	Ref.							
	Yes	−0.723	−2.024	0.577	0.275				

Operating theater	No	Ref.				Ref.			
	Yes	3.599	−1.229	8.427	0.144	1.915	−2.691	6.52	0.414

Intensive care unit	No	Ref.				Ref.			
	Yes	1.475	−0.395	3.344	0.122	0.763	−1.325	2.852	0.473

Pediatric	No	Ref.							
	Yes	0.309	−2.212	2.829	0.81				

Primary care	No	Ref.							
	Yes	−0.822	−4.961	3.318	0.697				

Emergency department	No	Ref.							
	Yes	−0.586	−3.686	2.514	0.71				

Other	No	Ref.							
	Yes	0.886	−0.829	2.601	0.311				

Working hours per month	< 100 h	Ref.							
	101–200 h	0.957	−1.91	3.824	0.512				
	201–300 h	1.474	−1.589	4.538	0.345				
	> 301 h	−1.365	−6.036	3.305	0.566				

Employment contract	No	Ref.							
	Yes	−0.492	−2.845	1.86	0.681				

Individual nursing practice	No	Ref.							
	Yes	1.287	−1.159	3.733	0.302				

Contract	No	Ref.							
	Yes	−1.542	−4.098	1.014	0.236				

Civil contracts (mandate/commission)	No	Ref.							
	Yes	0.538	−0.839	1.915	0.443				

Other	No	Ref.							
	Yes	2.056	−4.756	8.867	0.553				

Net monthly income	< 230 EUR	Ref.							
	230–460 EUR	1.917	−6.836	10.669	0.667				
	461–700 EUR	3.25	−5.502	12.002	0.466				
	701–930 EUR	2.05	−5.167	9.267	0.577				
	931–1160 EUR	3.336	−3.635	10.306	0.348				
	1161–1400 EUR	4.365	−2.518	11.247	0.213				
	> 1400 EUR	4.316	−2.523	11.154	0.216				

Salary satisfaction	Yes	Ref.				Ref.			
	No	−4.213	−5.476	−2.95	< 0.001^∗^	−3.828	−5.107	−2.549	< 0.001^∗^
	Undecided	−1.592	−3.706	0.523	0.14	−1.043	−3.147	1.061	0.331

Stress caused by Russia's invasion of Ukraine		−0.152	−0.218	−0.087	< 0.001^∗^	−0.127	−0.19	−0.063	< 0.001^∗^

^∗^Statistically significant relationship (*p* < 0.05).

**Table 4:** Predictors of optimism (LOT-R score).

Variable		Univariate models				Multivariate model			
		Parameter	95% CI		*p*	Parameter	95% CI		*p*
Sex	Female	Ref.							
	Male	−1.02	−3.067	1.027	0.328				

Age	< 30 years	Ref.				Ref.			
	31–40 years	1.105	−0.205	2.414	0.098	1.017	−0.275	2.308	0.122
	41–50 years	0.979	−0.137	2.095	0.085	0.733	−0.418	1.885	0.211
	51–60 years	0.807	−0.287	1.902	0.148	0.839	−0.33	2.007	0.159
	> 60 years	1.334	−0.719	3.387	0.202	0.979	−1.096	3.053	0.354

Education	Medical high school	Ref.				Ref.			
	Medical vocational school	−0.887	−2.526	0.752	0.288	−1.39	−3.019	0.239	0.094
	Bachelor's in nursing/midwifery	0.177	−1.038	1.391	0.775	0.089	−1.119	1.297	0.885
	Master's in nursing/midwifery	0.647	−0.533	1.827	0.282	0.535	−0.698	1.767	0.394
	Other	3.91	0.8	7.02	0.014^∗^	3.797	0.747	6.847	0.015^∗^

Qualification courses	No	Ref.							
	Yes	0.09	−0.65	0.829	0.812				

Specialist courses	No	Ref.							
	Yes	−0.333	−1.088	0.422	0.387				

Specialization	No	Ref.				Ref.			
	Yes	0.962	0.216	1.708	0.012^∗^	0.485	−0.297	1.268	0.224

Place of residence	Rural area	Ref.							
	City ≤ 50,000	−0.515	−1.486	0.456	0.298				
	City 50,001–100,000	−0.606	−1.842	0.629	0.335				
	City 100,001–250,000	−0.02	−1.414	1.374	0.977				
	City > 250,000	−0.478	−1.446	0.491	0.333				

Marital status	Single	Ref.							
	In a relationship	−0.121	−1.082	0.839	0.804				
	Widowed	−0.892	−3.31	1.527	0.469				

Profession	Nurse	Ref.							
	Midwife	−0.744	−1.89	0.402	0.203				

Number of jobs	1	Ref.				Ref.			
	2	−0.27	−1.085	0.545	0.515	−0.641	−1.49	0.208	0.139
	3	−0.759	−1.977	0.459	0.221	−1.345	−2.654	−0.037	0.044^∗^
	4	−0.655	−3.309	1.999	0.628	−0.796	−3.392	1.799	0.547
	5	0.501	−3.03	4.032	0.781	0.245	−3.195	3.686	0.889
	More than 5	−1.247	−2.836	0.341	0.124	−0.608	−2.179	0.962	0.447

Outpatient care	No	Ref.							
	Yes	−0.199	−0.948	0.55	0.602				

Inpatient care	No	Ref.				Ref.			
	Yes	−0.55	−1.335	0.234	0.169	−0.359	−1.148	0.43	0.372

Nursing home	No	Ref.							
	Yes	1.374	−1.119	3.867	0.279				

Long-term care	No	Ref.				Ref.			
	Yes	1.685	0.22	3.15	0.024^∗^	2.167	0.681	3.652	0.004^∗^

Trade union organization	No	Ref.							
	Yes	−1.541	−4.746	1.665	0.345				

University or college	No	Ref.							
	Yes	0.238	−1.367	1.842	0.771				

Other	No	Ref.							
	Yes	−0.224	−1.614	1.166	0.752				

Work schedule	Single-shift	Ref.							
	Shift-based	−0.324	−1.052	0.404	0.383				

Average number of patients per shift	1–5	Ref.				Ref.			
	6–10	1.081	−0.062	2.225	0.064	1.174	0.049	2.298	0.041^∗^
	11–15	0.894	−0.345	2.133	0.157	1.639	0.405	2.874	0.009^∗^
	16–25	0.66	−0.512	1.832	0.269	1.058	−0.118	2.234	0.078
	26–35	0.034	−1.41	1.478	0.963	0.363	−1.09	1.817	0.624
	> 36	0.394	−0.872	1.66	0.541	0.946	−0.383	2.274	0.162

Years of experience	0–5 years	Ref.							
	6–10 years	0.75	−0.699	2.199	0.309				
	11–15 years	0.781	−0.652	2.214	0.285				
	16–20 years	0.119	−1.54	1.778	0.888				
	> 21 years	0.444	−0.617	1.505	0.411				

Conservative	No	Ref.				Ref.			
	Yes	−0.97	−1.719	−0.222	0.011^∗^	−0.909	−1.726	−0.092	0.029^∗^

Surgical	No	Ref.							
	Yes	−0.171	−0.921	0.578	0.654				

Operating theater	No	Ref.				Ref.			
	Yes	2.945	0.173	5.717	0.037^∗^	2.148	−0.542	4.838	0.117

Intensive care unit	No	Ref.							
	Yes	−0.387	−1.465	0.691	0.481				

Pediatric	No	Ref.							
	Yes	0.224	−1.226	1.675	0.761				

Primary care	No	Ref.							
	Yes	1.359	−1.021	3.738	0.262				

Emergency department	No	Ref.							
	Yes	−0.997	−2.779	0.785	0.272				

Other	No	Ref.				Ref.			
	Yes	0.78	−0.205	1.766	0.12	0.189	−0.836	1.215	0.717

Working hours per month	< 100 h	Ref.				Ref.			
	101–200 h	−0.37	−2.02	1.28	0.659	−0.932	−2.54	0.675	0.255
	201–300 h	−0.544	−2.307	1.219	0.545	−0.86	−2.63	0.909	0.34
	> 301 h	−2.061	−4.748	0.627	0.133	−1.945	−4.647	0.756	0.158

Employment contract	No	Ref.							
	Yes	0.083	−1.271	1.437	0.904				

Individual nursing practice	No	Ref.							
	Yes	0.221	−1.188	1.63	0.758				

Contract	No	Ref.							
	Yes	−0.414	−1.887	1.058	0.581				

Civil contracts (mandate/commission)	No	Ref.							
	Yes	−0.415	−1.206	0.377	0.304				

Other	No	Ref.				Ref.			
	Yes	3.424	−0.485	7.333	0.086	2.337	−1.477	6.152	0.229

Net monthly income	< 230 EUR	Ref.							
	230–460 EUR	−1	−6.036	4.036	0.697				
	461–700 EUR	0.5	−4.536	5.536	0.845				
	701–930 EUR	−0.533	−4.686	3.619	0.801				
	931–1160 EUR	−0.314	−4.325	3.696	0.878				
	1161–1400 EUR	0.378	−3.582	4.338	0.851				
	> 1400 EUR	0.702	−3.233	4.637	0.726				

Salary satisfaction	Yes	Ref.				Ref.			
	No	−1.47	−2.214	−0.727	< 0.001^∗^	−1.433	−2.197	−0.669	< 0.001^∗^
	Undecided	−2.181	−3.426	−0.936	0.001^∗^	−2.105	−3.347	−0.863	0.001^∗^

Stress caused by Russia's invasion of Ukraine			−0.12	−0.045	< 0.001^∗^	−0.063	−0.101	−0.025	0.001^∗^

^∗^Statistically significant relationship (*p* < 0.05).

## Data Availability

The data that support the findings of this study are available from the corresponding author upon reasonable request.
